# Novel Set-Up for Low-Disturbance Sampling of Volatile and Non-volatile Compounds from Plant Roots

**DOI:** 10.1007/s10886-015-0559-9

**Published:** 2015-03-22

**Authors:** Elisabeth J. Eilers, Gerhard Pauls, Matthias C. Rillig, Bill S. Hansson, Monika Hilker, Andreas Reinecke

**Affiliations:** 1 Department of Applied Zoology/Animal Ecology, Freie Universität Berlin, Haderslebener Straße 9, 12163 Berlin, Germany; 2 Department of Evolutionary Neuroethology, Max-Planck-Institute for Chemical Ecology, Hans-Knöll-Straße 8, 07745 Jena, Germany; 3 Department of Bioorganic Chemistry, Max-Planck-Institute for Chemical Ecology, Hans-Knöll-Straße 8, 07745 Jena, Germany; 4 Department of Plant Ecology, Freie Universität Berlin, Altensteinstraße 6, 14195 Berlin, Germany; 5 Berlin-Brandenburg Institute of Advanced Biodiversity Research (BBIB), D-14195 Berlin, Germany; 6 Present Address: Department of Behavioural Ecology and Evolutionary Genetics, Max Planck Institute for Ornithology, Eberhard-Gwinner-Str. 7, 82319 Seewiesen, Germany

**Keywords:** Root exudates, Gas chromatography/mass spectrometry, Root volatiles, Sugars, Soil substrates

## Abstract

Most studies on rhizosphere chemicals are carried out in substrate-free set-ups or in artificial substrates using sampling methods that require an air flow and may thus cause disturbance to the rhizosphere. Our study aimed to develop a simplified and inexpensive system that allows analysis of rhizosphere chemicals at experimentally less disturbed conditions. We designed a mesocosm in which volatile rhizosphere chemicals were sampled passively (by diffusion) without air- and water flow on polydimethylsiloxane-(PDMS) tubes. Dandelion (*Taraxacum* sect. *ruderalia*) was used as model plant; roots were left undamaged. Fifteen volatiles were retrieved from the sorptive material by thermal desorption for analysis by gas chromatography/mass spectrometry (GC/MS). Furthermore, three sugars were collected from the rhizosphere substrate by aqueous extraction and derivatized prior to GC/MS analysis. In order to study how the quantity of detected rhizosphere compounds depends on the type of soil or substrate, we determined the matrix-dependent recovery of synthetic rhizosphere chemicals. Furthermore, we compared sorption of volatiles on PDMS tubes with and without direct contact to the substrate. The results show that the newly designed mesocosm is suitable for low-invasive extraction of volatile and non-volatile compounds from rhizospheres. We further highlight how strongly the type of substrate and contact of PDMS tubes to the substrate affect the detectability of compounds from rhizospheres.

## Introduction

Plant root-released volatile and non-volatile chemicals transmit information to other plants or edaphon organisms (Badri and Vivanco 2009; Bais et al. 2006; Rasmann et al. 2005; Van Tol et al. 2001; Wenke et al. 2010). So far, technical limitations have rendered analyses of rhizosphere compound blends difficult, particularly under natural conditions or in field studies. Important methodological parameters for analysis of rhizochemicals include the way roots are kept prior to analysis, the sampling technique, and the (ad)sorbent used.

Sophisticated radioactive or stable isotope labeling techniques, applicable even in field studies, have delivered comprehensive knowledge on carbon fluxes (e.g., carbohydrate transfer from plant roots to mycorrhizal fungi) in soil (Derrien et al. 2004; Johnson et al. 2002, 2005). Such labeling techniques, however, require complex and expensive experimental setups and often destructive processing of test plants. Other less complex and less costly studies on rhizochemicals apply methods that affect the release of root chemicals since the roots are not kept in a (close-to) natural way. For example, (i) extraction by solutions in which excavated plant roots are dipped (Gransee and Wittenmayer 2000; Ström et al. 1994), (ii) extraction from roots of hydroponic-grown plants (mostly sterile and hypoxic conditions) (Cieslinski et al. 1997; Ling et al. 2013; Neumann and Romheld 1999; Pramanik et al. 2000), or (iii) extraction from root systems of plants grown in agar, soda-glass beads or on filter paper (Eldhuset et al. 2007; Wang and Bergeson 1974). Root-derived compounds further have been analyzed from cut roots (Steeghs et al. 2004), frozen and sliced root mats (Gahoonia and Nielsen 1991), or extracts of pulverized roots (Rasmann and Turlings 2007).

Dynamic (active) sampling techniques for rhizochemicals use air- or water flow around the (often substrate-free) plant roots and through an adsorbent (e.g., Ali et al. 2010; Jassbi et al. 2010; Rasmann et al. 2011). Such dynamic sampling techniques are suitable and most frequently used to analyze plant volatiles aboveground (Tholl et al. 2006). However, air- or water flow around the plant roots is problematic due to the risk of drought or flooding. These methods may facilitate enhanced or diminished root exudation and oxygenation of trapped compounds. Again, the disturbance to the rhizosphere may be so drastic that the plants cannot be used for further experiments. Static (passive) chemical sampling methods (without air- or water flow) are based on diffusion of compounds into a substrate or sorbent. Widely used passive sampling techniques are solid phase microextraction (SPME) and stir-bar sorptive extraction (SBSE) (Baltussen et al. 1999).

Numerous adsorbents that bind analytes to their adsorptive sites and sorbents into which analytes are diffused and retained are commercially available (Baltussen et al. 1999). Woolfenden (2010) outlines a wide range of factors that need consideration when selecting an (ad)sorbent; sampling and desorption efficiency of the (ad)sorbent as well as artifact risks are parameters that significantly determine the quality of analysis. Well-known (ad)sorbents range from activated alumina, silica, zeolithes (aluminosilicates) to octadecyl-modified silica, activated carbon, and synthetic polymeric materials, e.g., Tenax TA consisting of 2,6-diphenylene-oxide polymer resin, or polydimethylsiloxane (PDMS).

Passive sampling of compounds on sorptive tubes based on polydimethylsiloxane (PDMS, silicone) has been accomplished successfully in the past and allows sampling of volatiles of widely divergent polarity and volatility (individually or in complex mixtures), and even those with low emission rates (Bartelt 1997; Nyasembe and Torto 2014; Tholl et al. 2006). Polydimethylsiloxane (PDMS) tubes are robust, easy to handle, and can be cut with scissors or blades into pieces of desired length. Furthermore, the tubes are inexpensive, and due to their size and hollowness, they provide a larger active surface than the active surface of a PDMS-covered fiber (Kallenbach et al. 2014). Sampling on PDMS tubes in soil or water is commonly combined with liquid chromatography, i.e., HPLC (Mohney et al. 2009; O’Hara 2009; Van Pinxteren et al. 2009; Weidenhamer et al. 2009, and references therein), and has, been applied e.g., to study the transport of allelopathic substances through mycorrhizal networks (Barto et al. 2011). Weidenhamer et al. (2009) used a similar method for collection of rhizosphere chemicals; they showed that PDMS tubes inserted into soil (solid phase root zone extraction; SPRE) were more efficient in collecting allelopathic rhizosphere compounds than any other tested (ad)sorbent. Sampling on PDMS tubing also may be combined with gas chromatography (GC). As the trapped compounds are not eluted with solvents, but thermally desorbed prior subjection to gas chromatography, a noise-free baseline can be obtained (Bartelt 1997). Recently, Kallenbach et al. (2014) presented a high-throughput technique for the collection of volatiles from aerial plant parts in field experiments using PDMS tubes.

This study aimed (i) to develop a set-up that allows inexpensive, simple, and reliable collection of volatiles (using PDMS tubing for sampling) as well as non-volatile hydrophilic compounds (using aqueous substrate extraction) from a rhizosphere that is not exposed to disturbing conditions like air- or water flow through the root zone, and (ii) to elucidate conditions at which rhizosphere compounds adsorb optimally on PDMS tubes.

A rhizosphere mesocosm was designed from which volatiles were extracted on PDMS tubes without root damage, air- or water flow. We focused on the analysis of rhizosphere volatiles because of their ecological relevance in root-associated food webs (Hiltpold et al. 2010; Robert et al. 2012; Van Tol et al. 2001; Weissteiner et al. 2012). In order to evaluate the potential use of the mesocosm for aqueous extraction of polar, water-soluble compounds, we also analyzed plant root-derived sugars as one example of this type of compound. We focused on sugars because of their quantitative dominance in rhizospheres (Azaizeh et al. 1995). For analysis of sugars, minor amounts of substrate encasing the roots were harvested, and extracted sugars were derivatized prior to GC/MS analysis.

Rhizosphere compounds belong to diverse chemical classes with divergent affinity to PDMS and to the substrate or soil. We analyzed how well PDMS tubes compete with different adsorbing substrates by determining recovery rates of synthetic reference compounds that have been identified previously in natural rhizospheres. We further tested whether protecting PDMS tubes against clogging by soil particles could enhance the recovery rates of volatiles.

## Methods and Materials

### Plants

The ubiquitous (Stewart-Wade et al. 2002) and pharmacologically relevant (Williams et al. 1996) ruderal plant dandelion (*Taraxacum* sect. *ruderalia*, Kirschner, Øllgaard *et* Štěpánek) was chosen to test the suitability of the newly developed rhizosphere mesocosm for analysis of rhizosphere chemistry. Dandelion seeds (Treppens & Co Samen GmbH, Berlin) were surface-sterilized as described by Krügel et al. (2002). Seedlings were placed individually in 100 ml sand-filled plastic flower pots. All plants were watered with nutrient solution modified from Arnon and Hoagland (1940), see Reinecke et al. (2008). Young plants were grown in a climate chamber at 22 °C, 70 % r.h., L:D 16:8 h. When plants were 5-wk-old, they were transplanted into rhizosphere mesocosms (see below).

### Novel Rhizosphere Mesocosm

**Fig. 1:**
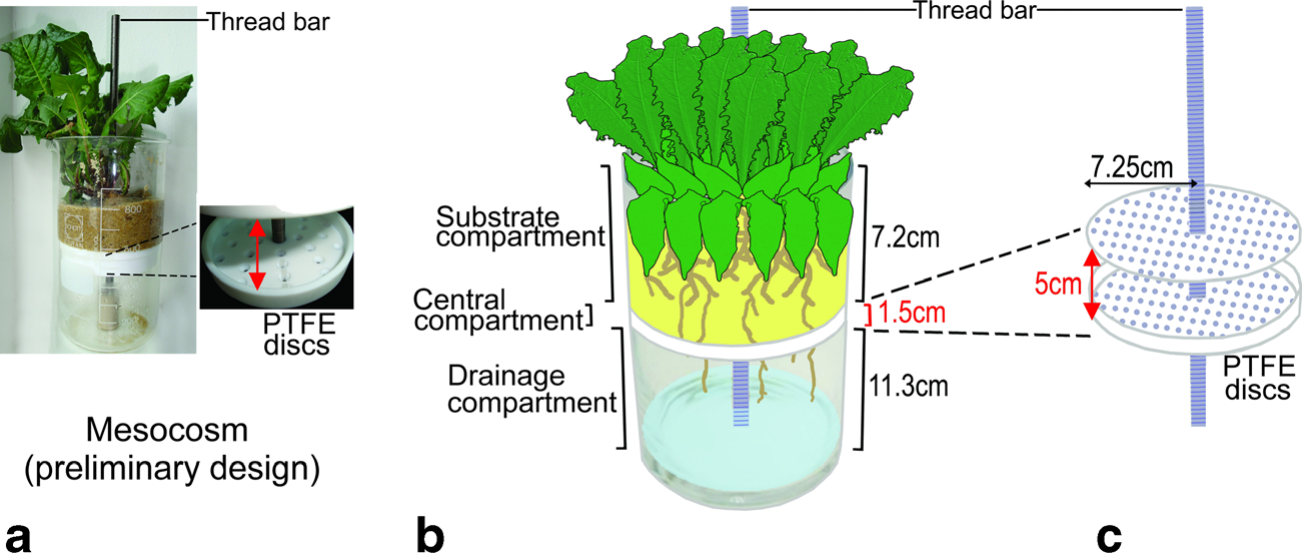
Rhizosphere mesocosm for collection of root-derived volatile and water-soluble compounds. **a** The size of the mesocosm may be adapted to the number and size of plants: The mesocosm shown in this photo basically consisted of the same components as the mesocosm used in the experiments (except for the fleece in the substrate compartment), but a 1 L Schott beaker glass replaced the glass vessel. **b** The mesocosm consisted of a substrate compartment (filled with plant roots and sand), a central compartment (filled with humid air, buffer zone between substrate and drainage compartment), and a drainage compartment (containing leached aqueous nutrient solution that was not taken up by the plants and accumulated here over 5 d; approx. 5 mm, *V* = 80 ml solution in total). The plant roots grew through the perforated polytetrafluoroethylene (PTFE) discs (124 holes, diam 2 mm), which were mounted on a stainless steel thread bar (length: 25 cm, diam 1 cm). Due to static (passive) volatile collection on polydimethylsiloxane (PDMS) tubes, no artificial air flow was required. **c** The thread bar, which carried the PTFE discs, allowed gentle removal of rhizospheres prior to insertion of PDMS tubes for sampling of volatiles. Fleece surrounding the substrate minimized friction between the plant roots, substrate, and the glass vessel when removing the plant with root ball from the substrate compartment for insertion of PDMS tubes in the central and/or drainage compartment

The rhizosphere mesocosms consisted of silanized glass vessels (Fig. 1a), containing two horizontally stacked perforated polytetrafluoroethylene (PTFE) discs (124 holes of 2 mm diam) mounted on a stainless steel thread bar (Fig. 1b). The size of holes in the discs was adjusted to the size of the roots of the plants. Thus, roots could grow through the discs without getting stuck. The discs were designed similar to desiccator plates, but the lower disc had a spacer rim of 1.5 cm height. Each rhizosphere mesocosm was divided into three compartments by the discs: (i) a sand-filled compartment (1.2 L, top), (ii) a central compartment between the discs (250 ml, height = 1.5 cm), and (iii) a drainage compartment (2.1 L, bottom; Fig. 1b). Due to the high moisture content of both adjacent compartments, the central compartment contained air of high humidity, and thus, roots reaching this compartment were not exposed to drought. A shading aluminum foil cover was wrapped around the outside of each mesocosm and prevented algal growth inside the glass vessels. The inside of each mesocosm was lined with clean polyester fleece, which provided a barrier between roots, substrate, and glass. Thus, roots could not attach to the glass walls, and the entire plant and root ball could gently be removed from the mesocosm and inserted back again when necessary without damaging the roots. Non-destructive removal of the root ball from the mesocosms was essential for sampling of rhizosphere volatiles (see section ‘*Rhizosphere Mesocosm: Extraction of Root-Derived Volatiles’*).

Five-wk-old plants were transplanted into sand-filled rhizosphere mesocosms. Groups of 10 plants each were combined in order to compensate for differences among individuals. Plants were grown for an additional period of 3.5 wk in the mesocosm prior to insertion of PDMS tubing pieces for volatile sampling. Sampling of PDMS tubing pieces for chemical analysis of volatiles and collection of substrate samples for chemical analysis of sugars was conducted when the plants were 9-wk-old. In total, rhizosphere chemicals of six mesocosms, i.e., groups of 10 plants each were analyzed. The conditions at which plants were kept in the mesocosms were 22 °C, 70 % r.h., L:D 16:8 h, and 791.37 ± 31 μmol∙s^−1^∙m^−2^ PAR light intensity. Plants in the rhizosphere mesocosms were irrigated daily until a defined drainage from the substrate was observed in the drainage compartment (approx. 5 mm, i.e., 80 ml in total collected over 5 d). The leached solution was not pumped back to the rhizosphere. Volatile rhizosphere compounds were collected in all mesocosm compartments on PDMS tubes. Sugars were extracted from the substrate in the upper (substrate) compartment and lower drainage compartment.

### Rhizosphere Mesocosm: Extraction of Root-Derived Volatiles

Volatiles were collected on PDMS tubes (inner diam: 1.5 mm, o.d.: 2.3 mm, length: 3 mm, Reichelt Chemietechnik, Germany). PDMS bulk tubes were cut into pieces of 3 mm using a custom made cutting mask with razor blades in guiding rails at 3 mm distance. Tube pieces were cleaned for 8 h in 4:1 (*v/v*) acetonitrile: methanol, and afterwards heated to 230 °C for 12 h under nitrogen flow (filtered on activated charcoal) in a tube conditioner (TC 2, Gerstel, Mülheim, Germany) prior to analysis. As it was unknown from which compartment the volatiles could be extracted most efficiently, we collected volatiles from all three compartments (substrate, central, and drainage) of the rhizosphere mesocosms (Fig. 1a). Three tube pieces in one compartment were regarded as technical replicates, and the average of these technical replicates was considered one biological replicate. We analyzed compartments of six mesocosms (i.e., six biological replicates). For control, volatiles were collected from six identically treated but plant-free mesocosms. In the upper, substrate-filled compartment, PDMS tube pieces were placed at 3–4 cm depth into plant rhizospheres or substrate-filled control compartments of mesocosms. For insertion of the PDMS tubes into the central and drainage compartments, the intact plants with root balls were gently withdrawn from the rhizosphere compartments together with the PTFE discs by pulling the thread bar (Fig. 1b) and overlapping fleece. The plants and root ball remained on the upper PTFE disc, and the screw holding the lower PTFE disc was moved downward to obtain a 5 cm slot for insertion and removal of PDMS tubes into the central compartments (between the two PTFE discs). Although plant roots sporadically grew through the holes in the perforated discs, they were not ruptured nor abraded by the PTFE discs or the glass vessel during the insertion or removal procedure.

In a preliminary experiment, different extraction durations (12, 24, 48, 96, and 144 h) were assessed by sampling volatiles from three dandelion mesocosms, each containing 10 plants (*N* = 3). The proportions of total peak areas of plant compounds, i.e., compounds not present in substrate controls were: 12.9 ± 2.4 % (12 h), 10.2 ± 2.2 % (24 h), 32.6 ± 10 % (48 h), 100 % (96 h), 83.7 ± 17.7 % (144 h) relative to the highest measured peak area for each compound (mean ± s.d.). As sampling efficiency was highest for both 96 and 144 h and did not significantly differ between these durations (*P* > 0.05, Wilcoxon signed-rank test), we chose an intermediate sampling duration of 120 h for further analyses. After sampling, PDMS tubes were removed from rhizosphere mesocosms and stored individually in 1.5 ml screw-cap glass vials at −20 °C until analysis.

### Rhizosphere Mesocosm: Extraction of Root-Derived Sugars

For collection of a rhizosphere substrate sample for sugar extraction, the intact plants with root balls were gently removed together with the substrate by withdrawing the PTFE discs, thread bar, and overlapping fleece from the mesocosms (Fig. 1). Then, two substrate samples (two technical replicates, each 15 ml) were collected from each substrate compartment (*N =* 6 mesocosms). These samples included only loose substrate that had encased plant roots. The plant roots themselves, however, remained intact during substrate removal. Plants were not used again. In addition to samples from the substrate compartment, we also sampled the leached nutrient solution collected over 5 d in the drainage compartment (two technical replicates of 15 ml per sample; Fig. 1). Remaining leached nutrient solution was discarded, and mesocosms were not sampled again for sugars. Samples of substrate and leached nutrient solution were kept on ice during collection (approx. 15 min) and later stored at −80 °C until extraction and analysis. The (root-free) substrate samples were extracted by adding 15 ml distilled water to 15 ml substrate. The mixture was sonicated for 10 min. The filtrate was obtained by using a suction filter, and it was cooled on ice. After repeating this procedure, approx. 25 ml filtrate per sample were obtained, which were immediately frozen at −80 °C, lyophilized to dryness, and dissolved in pyridine to 2 μg μl^−1^. The samples taken from the drainage compartment (15 m) also were lyophilized to dryness and dissolved in pyridine to 2 μg μl^−1^.

### Recovery of Rhizosphere Compounds from Different Substrates in Vials: General Procedure

To determine substrate specific recovery rates of reference compounds, we used substrate-filled glass vials (40 ml screw top, Supelco, Sigma-Aldrich) into which PDMS tubes were inserted (for volatile sampling), or from which the substrates were extracted (for sampling of sugars). Recovery rates were compared for distinct quantities of commercially available rhizosphere compounds that were added to water, sand, vermiculite, a fraction of processed (heated and sieved) soil (Tables 1 and 2), and unprocessed soil (Table 3) (see below for details on the substrates and tested compounds). Glass vials (40 ml screw top, Supelco, Sigma-Aldrich) were filled with 21 ml water or 25 ml dry substrate (see below) supplemented with 21 ml water. The non-dried field soil was supplemented by 10 ml water to reach a comparable moisture level. Commercially available compounds were added to these substrates in distinct quantities (see below).

**Table 1:** Determination of recovery rates (%) of standard volatiles applied to different substrates in a glass vial and sampled by polydimethylsiloxane (PDMS) tubes with *contact* to substrate or by gauze-*protected* PDMS tubes

Compound^a^	Setup	Substrates			
		Water	Sand	Vermiculite	Processed soil^b^
butyl acetate^c^	contact PDMS	0.3 ± 0.0 **(C)**	1.2 ± 0.2 **(B)**	2.8 ± 0.4 **(A)**	1.3 ± 0.1 (B*)
	protected PDMS	2.2 ± 0.6 (B*)	1.9 ± 0.4 **(B)**	5.4 ± 1.7 (A*)	0.7 ± 0.3 (**C)**
1-hexanol^c^	contact PDMS	0.0 ± 0.0 **(C)**	0.4 ± 0.1 **(A)**	0.2 ± 0.0 **(B)**	0.0 ± 0.0 **(C)**
	protected PDMS	1.4 ± 0.4 (A*)	1.9 ± 0.4 (A*)	1.6 ± 0.5 (A*)	0.2 ± 0.1 **(A)**
α-pinene^c^	contact PDMS	5.0 ± 0.5 **(C)**	12.0 ± 1.4 **(B)**	15.2 ± 1.2 **(A)**	7.2 ± 0.8 **(C)**
	protected PDMS	19.2 ± 7.4 (B*)	9.7 ± 1.1 **(BC)**	32.9 ± 5.6 (A*)	6.7 ± 0.8 **(C)**
benzaldehyde^c^	contact PDMS	0.6 ± 0.1 **(C)**	5.1 ± 0.9 (A*)	2.5 ± 0.4 **(B)**	5.1 ± 0.8 (A*)
	protected PDMS	1.3 ± 0.2 (A*)	2.4 ± 1.1 **(A)**	2.3 ± 0.3 **(A)**	2.6 ± 0.4 **(B)**
hexyl acetate^c^	contact PDMS	0.4 ± 0.1 **(B)**	0.9 ± 0.1 **(A)**	0.9 ± 0.1 **(A)**	0.3 ± 0.0 **(B)**
	protected PDMS	5.1 ± 1.6 (B*)	1.6 ± 0.4 (C*)	9.3 ± 1.7 (A*)	0.2 ± 0.0 **(D)**
linalool^c^	contact PDMS	2.4 ± 0.3 **(B)**	6.1 ± 1.0 **(A)**	2.6 ± 0.3 **(B)**	6.9 ± 0.8 **(A)**
	protected PDMS	6.6 ± 0.3 (AB*)	7.4 ± 0.3 **(A)**	7.9 ± 0.3 (A*)	5.9 ± 0.5 **(B)**
methyl salicylate^d^	contact PDMS	0.1 ± 0.0 **(C)**	0.7 ± 0.1 **(A)**	0.1 ± 0.0 **(C)**	0.2 ± 0.0 **(B)**
	protected PDMS	1.8 ± 0.2 (B*)	1.9 ± 0.2 (B*)	2.8 ± 0.4 (A*)	0.4 ± 0.2 **(C)**
cinnamal^c^	contact PDMS	1.2 ± 0.1 **(C)**	3.3 ± 0.6 **(A)**	1.6 ± 0.2 **(B)**	3.1 ± 0.5 **(AB)**
	protected PDMS	2.6 ± 0.4 (A*)	4.3 ± 0.6 **(A)**	1.8 ± 0.3 **(A)**	4.6 ± 0.4 **(B)**
β-elemene^d^	contact PDMS	45.6 ± 3.6 **(A)**	5.9 ± 1.4 **(B)**	11.4 ± 1.5 **(B)**	8.3 ± 1.6 **(B)**
	protected PDMS	43.7 ± 2.9 **(A)**	7.8 ± 0.4 **(C)**	18.3 ± 1.5 (B*)	8.5 ± 1.8 **(C)**
β-farnesene^d^	contact PDMS	13.9 ± 1.0 **(A)**	9.1 ± 0.7 **(B)**	15.0 ± 1.5 **(A)**	0.7 ± 0.2 **(C)**
	protected PDMS	12.8 ± 1.3 **(A)**	9.6 ± 0.9 **(A)**	14.6 ± 0.8 **(A)**	2.1 ± 0.6 (B*)
α-farnesene^d^	contact PDMS	22.3 ± 3.1 **(B)**	21.5 ± 2.0 **(B)**	36.0 ± 3.4 **(A)**	0.9 ± 0.4 **(C)**
	protected PDMS	19.2 ± 5.9 **(B)**	18.7 ± 2.3 **(B)**	35.3 ± 8.7 **(A)**	2.1 ± 0.6 (C*)
farnesyl acetate^d^	contact PDMS	0.1 ± 0.0 **(C)**	2.5 ± 0.3 **(A)**	0.4 ± 0.1 **(B)**	2.6 ± 0.3 **(A)**
	protected PDMS	3.5 ± 1.4 (A*)	3.5 ± 1.2 **(A)**	0.4 ± 0.1 **(B)**	1.8 ± 0.6 **(AB)**

^a^ Compounds ordered by KI (Kovats retention index); 100 ng of each volatile were applied per vial; 100 % = peak area of 100 ng of each compound directly subjected to GC/MS analysis (mean ± SD, *N* = 4)

^b^ Sieved and heated soil

^c^ Compounds that have been studied with respect to interactions between dandelion roots and a rhizophagous insect (Eilers et al. 2012)

^d^ Compounds that were also detected in dandelion rhizospheres (compare Table 4)

* Asterisks indicate significant differences between contact and protected PDMS within one compound and substrate category at *P* ≤ 0.05 (Wilcoxon signed-rank test); different letters indicate significant differences between substrates at *P* ≤ 0.05 (Kruskal-Wallis *H-*test and *post-hoc* Mann–Whitney *U*-tests with Bonferroni correction)

**Table 2:** Recovery rates (%) of standard sugars applied to different substrates in a glass vial and sampled by aqueous extraction

Compound^a^	Substrates			
	Water	Sand	Vermiculite	Processed soil fraction^b^
arabinose (C5)^c^	42.2 ± 11.9 **(A)** ^e^	15.3 ± 5.7 **(B)**	3.8 ± 1.2 **(C)**	1.5 ± 0.3 **(C)**
xylose (C5)^c^	86.6 ± 7.1 **(A)**	12.4 ± 1.3 **(B)**	4.2 ± 0.7 **(C)**	1.6 ± 0.2 **(D)**
mannose (C6)^c^	58.7 ± 8.1 **(A)**	17.4 ± 2.4 **(B)**	3.6 ± 0.8 **(C)**	3.2 ± 0.4 **(C)**
fructose (C6)^d^	46.4 ± 7.2 **(A)**	10.5 ± 0.5 **(B)**	2.6 ± 0.5 **(C)**	1.2 ± 0.5 **(D)**
glucose (C6)^d^	70.7 ± 10.4 **(A)**	16.6 ± 2.9 **(B)**	4.4 ± 0.4 **(C)**	2.2 ± 0.1 **(D)**
sucrose (C12)^d^	25.3 ± 8 **(A)**	13.5 ± 1.8 **(B)**	2.6 ± 0.5 **(C)**	1.6 ± 0.3 **(C)**
maltose (C12)^c^	85.8 ± 7.6 **(A)**	6.3 ± 0.1 **(B)**	1.1 ± 0.3 **(C)**	1.0 ± 0.1 **(C)**

^a^ Ordered by KI (Kovats retention index); 10 μg of each sugar was applied per vial. 100 % = peak area of 100 ng of each compound directly subjected to GC/MS analysis (mean ± SD, *N* = 4)

^b^ Sieved and heated soil

^c^ Compounds that were also detected in rhizospheres of other plants than dandelion (Dennis et al. 2010)

^d^ Compounds that were also detected in dandelion rhizospheres (compare Table 4)

^e^ Different letters indicate significant differences between substrates at *P* ≤ 0.05 (Kruskal-Wallis *H-*test and *post-hoc* Mann–Whitney *U*-tests with Bonferroni correction)

**Table 3:** Recovery rates (%) and total amounts of volatiles applied to unprocessed soil and sampled by polydimethylsiloxane (PDMS) tubes with contact to soil

Compound^a^		Amounts applied to unprocessed field soil			
		100 ng^b^	500 ng	1 μg	5 μg
Volatiles	%	ng	ng	ng	ng
butyl acetate^c^	0.0 ± 0.0	0.0 ± 0.0	0.1 ± 0.0	0.0 ± 0.0	0.0 ± 0.0
1-hexanol^c^	0.0 ± 0.0	0.0 ± 0.0	0.1 ± 0.0	0.1 ± 0.1	1.1 ± 0.5
α-pinene^c^	0.2 ± 0.0	0.2 ± 0.1	14.9 ± 1.2	32.5 ± 2.6	61.2 ± 4.1
benzaldehyde^c^	0.0 ± 0.1	0.0 ± 0.0	0.1 ± 0.0	2.6 ± 0.2	7.6 ± 0.8
hexyl acetate^c^	0.0 ± 0.2	0.1 ± 0.0	0.1 ± 0.0	0.0 ± 0.0	0.0 ± 0.0
linalool^c^	0.0 ± 0.1	0.0 ± 0.0	7.8 ± 0.6	10.1 ± 0.8	5.5 ± 0.6
methyl salicylate^d^	0.2 ± 0.1	0.1 ± 0.0	1.7 ± 0.4	2.2 ± 0.2	44.0 ± 3.6
cinnamal^c^	0.3 ± 0.2	0.1 ± 0.0	2.2 ± 0.3	4.1 ± 0.4	12.1 ± 0.9
β-elemene^d^	0.0 ± 0.0	0.0 ± 0.0	1.2 ± 0.3	1.3 ± 0.4	2.6 ± 0.3
β-farnesene^d^	0.0 ± 0.0	0.0 ± 0.0	1.6 ± 0.2	1.4 ± 0.1	2.5 ± 0.4
α-farnesene^d^	0.0 ± 0.0	0.0 ± 0.0	0.5 ± 0.1	0.5 ± 0.1	1.0 ± 0.4
farnesyl acetate^d^	0.3 ± 0.2	0.1 ± 0.0	6.1 ± 0.5	7.4 ± 0.0	9.0 ± 1.4

^a^ Sorted by KI

^b^ 100 % = peak area of 100 ng of each compound directly subjected to GC/MS analysis (mean ± SD, *N* = 4)

^c^ Compounds that have been studied with respect to interactions between *T.* sect. *ruderalia* roots and a rhizophagous insect (Eilers et al. 2012)

^d^ Compounds that were also detected in dandelion rhizospheres (compare Table 4)

**Fig. 2:**
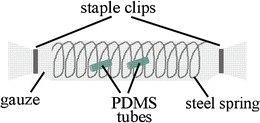
Gauze sheath for polydimethylsiloxane (PDMS) tubes in volatile recovery experiments with standard components. In order to evaluate the effect of contact between the polydimethylsiloxane (PDMS) tubes and the tested substrates (and water) on the recovery of applied standard volatiles, PDMS tubes were either placed directly into the matrix or enveloped in 100 μm PEEK™ (polyether ether ketone) gauze, as illustrated in this figure. A stainless steel spring was placed inside the gauze sheath to maintain a constant volume (0.69 cm^2^). Both ends of the gauze sheath were sealed with steel staple clips

The incubation time, i.e., the time lag between spiking of the test compounds into the substrate-filled vials and obtainment of samples for determination of recovery rates by GC-MS analysis was 1 h (volatiles) or 15 min (sugars). Volatiles were collected on PDMS tubes as described above for volatile collection in the dandelion mesocosm; three conditioned PDMS tube pieces (3 mm long) were inserted into each vial, either directly into the water or substrate, or protected by a polyetheretherketone (PEEK™) gauze sheath (100 μm mesh openings, 32 % open area, Sefar Peektex®, Sefar AG, Heiden, Switzerland). The gauze was closed with steel staple clips, and a steel spring provided for a constant volume of approx. 0.7 cm^2^ inside the gauze (Fig. 2). Sugars were extracted as described above (see section ‘*Rhizosphere mesocosm: Extraction of Root-Derived Sugars’*).

### Recovery of Rhizosphere Compounds from Different Substrates in Vials: The Substrates

We compared the recovery rates from water and the following substrates:

- Sand: pH (CaCl_2_) = 5.5, particle size ∅ = 0.4–0.8 mm, specific surface area (SSA): approx. 0.01–0.005 m^2^·g^−1^, Cation exchange capacity (CEC): 0 meq·100 g^−1^ (all particle size, SSA and CEC values according to Ahl et al. 2004; pH (CaCl_2_) was determined according to Kerschberger et al. 2000).
- Vermiculite: pH (CaCl_2_) = 6.92, cluster size ∅ = 2–3 mm, SSA: 60–80 m^2^ g^−1^, CEC: approx. 80 meq·100 g^−1^.
- Processed (sieved and heated) fraction of field soil: sandy clay loam, collected from a dandelion-rich meadow in Hessenthal, Bavaria, Germany (49°93′N, 9°26′O) in October 2010 and April 2011. The organic fraction was partly removed by sieving (1 mm) and heating to 200 °C for 3 h. After processing, the pH (CaCl_2_) was 5.9, indicating a humus content of 15 % (Kerschberger et al. 2000). Supposing that the composition of this sandy clay loam matches the known composition of this type of soil (25–30 % clay, 5–25 % silt, 55–60 % sand, and 15 % humus), the CEC is expected to range between 55–61 meq·100 g^−1^.
- Natural, untreated soil: sandy clay loam, collected from a dandelion-rich meadow in Berlin, Germany (52.45′N, 13.31′O) in March 2014 and used for experiments without further processing. The pH (CaCl_2_) was 6.5, indicating a humus content of 12–15 % (Kerschberger et al. 2000). Supposing that the composition of this untreated soil matches the known composition of this type of soil (30–35 % clay, 5–25 % silt, 45–50 % sand, and 12–15 % humus), the CEC is expected to range between 54–66 meq·100 g^−1^.

### Recovery of Rhizosphere Compounds from Different Substrates in Vials: The Test Compounds

The volatiles tested here were chosen because (i) they were detected in dandelion rhizospheres, or (ii) they are presumably relevant in an interaction between a rhizophagous insect (*Melolontha melolontha* larvae) and dandelion roots (Eilers et al. 2012). The sugars were chosen because they were identified in the rhizosphere of dandelion (glucose, fructose, sucrose) or have been described in root exudates of other plants (Dennis et al. 2010). The following synthetic volatiles were used: β-elemene (Aapin Chemicals Limited, Abingdon, Oxfordshire, UK), benzaldehyde, butyl and hexyl acetate, cinnamaldehyde (cinnamal), α- and β-farnesene, farnesyl acetate, 1-hexanol, linalool, methyl salicylate (all Sigma-Aldrich, Steinheim, Germany), α-pinene (Fluka, Steinheim, Germany). A mix of these compounds was dissolved in dichloromethane (100 ng μl^−1^ of each compound). A volume of 1 μl of the mixture was added to each vial filled with substrate as described above (*N =* 4 vials for each type of substrate or water) (Table 1). In natural, untreated soil, we additionally tested the recovery of higher amounts: 5 μl of the mixture (500 ng of each compound), 10 μl (1 μg of each compound), and 50 μl (5 μg of each compound) (Table 3). Spiked substrate and water samples were vortexed for 2 min to distribute the compounds evenly and expose them to the substrate particles. Afterwards, PDMS tubes were inserted and kept for 60 min before they were subjected to thermal desorption and GC/MS analysis.

The following sugars were included in recovery experiments (Table 2): sucrose, maltose, mannose (all Sigma Aldrich, Steinheim, Germany), glucose, fructose, arabinose, and xylose (all Roth, Karlsruhe, Germany). A mix of sugars was dissolved in water (10 μg ml^−1^). We added 20 ml water and 1 ml sugar solution to each vial (*N =* 4 vials for each type of substrate or water) and vortexed the mix intensively for 2 min to distribute the compounds in the matrix. Thereafter, the samples were kept for 15 min before the entire sample was extracted, lyophilized, derivatized, and analyzed by GC/MS.

### Analytical Procedures for Rhizosphere Chemicals in Mesocosms and Recovery Experiments

Analytical separation of volatiles and derivatized sugars was carried out using an Agilent 7890A gas chromatograph (GC) (Agilent Technologies; Waldbronn, Germany) connected to an Agilent 5975 C mass spectrometer (MS). The MS was operated in electron impact mode (70 eV). Helium was used as carrier gas (constant flow 1 ml min^−1^).

For analysis of volatiles, the instrument was equipped with a thermal desorption unit (TDU, Gerstel), coupled to a programmable temperature vaporization (PTV) injector unit (Gerstel, KAS 4). The volatiles were separated on an HP-5 ms column (30 m × 0.25 mm i.d. with 0.25 μm-film coating, Agilent technologies). After insertion of PDMS sampling tubes (one tube per analysis), the TDU temperature increased from 30 °C to 210 °C at a rate of 30 °C min^−1^ and was kept for 10 min (1 min initial delay time, total desorption period = 18 min). Thermally desorbed compounds were trapped in the N_2_-cooled injection unit at −50 °C. The GC run started by heating the injection system at a rate of 12 °C s^−1^ to 220 °C, kept for 5 min. The GC oven was kept at 40 °C for 5 min, heated at 5 °C min^−1^ to 260 °C, and kept for 7 min (total run time 56 min). Kovats retention time indices (KI) were calculated for each compound based on comparison of retention times to n-alkane standard compounds (C8-C20, 100 ng μl^−1^ in hexane, Supelco, Sigma-Aldrich).

The identification of volatiles was accomplished by comparison of KI and mass spectra to authentic reference compounds when available: β-elemene (Aapin Chemicals Limited), 2-ethyl-1-hexanol, methyl salicylate, 2-phenoxyethanol, β-farnesene, farnesyl acetate (all Sigma-Aldrich); α-selinene and β-selinene from celery (*Apium graveolens*; Hardt et al. 1995) as well as pethybrene and α-isocomene from butterbur (*Petasites hybridus*; Saritas et al. 2002), kindly provided by Stephan H. von Reuß, MPI CE Jena, Germany. The remaining compounds were identified tentatively by comparison of mass spectra and KI values with those available in the National Institute of Standards and Technology (NIST) library or in the MassFinder terpenoid library (Hochmuth, Hamburg, Germany). See Table 4 for information on compound identification.

**Table 4:** Estimation of absolute quantities of volatiles and sugars detected in rhizosphere mesocosms containing *Taraxacum* sect. *ruderalia* plants and sampled by polydimethylsiloxane (PDMS) tubes with direct contact to substrate (volatiles) or by aqueous extraction (sugars)

#	Ref.	Compound^a^	KI (HP-5 ms)	Amount (ng) (mean ± SD)
Volatiles				
1	♦	*2-ethyl-1-hexanol (likely a contamination)*	1032	156.2 ± 129.6
2	♦	methyl salicylate	1195	20.6 ± 47.5
3	♦	2-phenoxyethanol	1221	11.2 ± 24.7
4	♣	panaginsene	1335	Not quantified
5	♦	pethybrene	1377	52 ± 41.2
6	♣	african-2-ene	1385	Not quantified
7	♦	α-isocomene	1388	187.9 ± 218.5
8	♦	β-elemene	1398	7.1 ± 5.9
9		*m/z*: 119(100), 189(76), 161(70), 204(32), 91(24); M: 204	1447	Not quantified
10	♦	β-farnesene	1458	1.9 ± 3.1
11		*m/z:* 109(100), 110(56), 204(43), 79(41), 93(28); M: 204	1463	Not quantified
12	●	γ-selinene	1485	Not quantified
13	♦	β-selinene (eudesma-4(14),11-diene)	1493	48.1 ± 80.2
14	♦	α-selinene	1497	62.2 ± 95.4
15	♦	farnesyl acetate	1843	0.6 ± 1.85
Sugars				
16	♦	fructose	–	40.7 ± 48.6
17	♦	glucose	–	197 ± 143.3
18	♦	sucrose	–	279.9 ± 239.8

^a^ Extraction of compounds from the central compartment (volatiles) and substrate compartment (sugars) of a rhizosphere mesocosm with dandelion plants (*N* = 6 biological replicates, corresponding to 6 mesocosms containing groups of 10 plants each). No sugars were detected in the drainage compartment. All 15 volatiles have been found in the central compartment, whereas the substrate and drainage compartments contained only 12 and 8 of the compounds, respectively

●: Identification via NIST library (Mass spec. match >95 % and KI ± 5)

♣: Identification via MassFinder

♦: Compared to reference compound (Mass ± spec. match >95 % and KI ± 5)

For analysis of derivatized sugars, an HP-1 ms capillary column (30 m × 0.25 mm i.d. with 0.25 μm-film coating, Agilent Technologies) was used. The GC-injection port for liquid samples was kept at 240 °C and operated in splitless mode. The initial oven temperature of 60 °C was kept for 3 min and then increased at 4 °C min^−1^ to a final temperature of 300 °C kept for 1 min (total run time 64 min). Sugars were identified by comparison of mass spectra and retention times to those of authentic standards (all Roth, Karlsruhe, Germany). Lyophilized samples (extracted from rhizosphere substrate), dissolved in 50 μl pyridine were derivatized with 50 μl N,O-bis(tri-methylsilyl)trifluoroacetamide (BSTFA) supplemented with 1 % tri-methylsilyl chloride (Sigma-Aldrich, Steinheim, Germany). The samples were kept for 90 min at 37 °C on a thermo shaker. Furthermore, a volume of 900 μl pyridine was added to each sample prior to analysis (total volume = 1 ml, injection volume = 1 μl).

### Quantification of Compounds in Dandelion Mesocosm Rhizospheres

Prior comparison of relative quantities and estimation of absolute quantities of detected compounds in mesocosms (within compartments and between compartments), a standard response factor was calculated for each sample based on the peak area of an internal standard (r.f.: measured peak area of a standard in sample divided by the peak area of the same amount of standard directly subjected to GC/MS). For volatiles, 1-bromodecane (Sigma-Aldrich, Germany; 100 ng in 1 μl in dichloromethane, added directly onto the tube piece) was added to an extraction sample prior to analysis; for sugars, phenyl-β-D-glucoside (Fisher Scientific, Germany; 100 ng in 1 μl^−1^ pyridine) was added as internal standard prior to sample derivatization. The peak area of each compound was normalized (multiplied) by the standard response factor for each sample.

For estimation of absolute quantities of detected compounds, calibration curves of reference compounds (see section ‘*Analytical Procedures*’) were obtained: for volatiles at concentrations of 100, 50, 20, and 5 ng per μl, solved in dichloromethane and for sugars at concentrations of 0.2, 0.8, 1, 2, 5, and 10 μg per μl, solved in pyridine. Each measurement of reference compounds was repeated three times. For volatiles, one μl of the dilution was applied to a PDMS tube and for sugars, one μl of the dilution was derivatized and analyzed in the same way as the sugar samples obtained from substrates. Absolute quantities were estimated by comparison of peak areas in samples (corrected by the standard response factor, i.e., internal standard phenyl-β-D-glucoside) to peak areas from calibration curves obtained from reference compounds.

### Determination of Recovery Rates from Rhizosphere Compounds Added to Substrates in Vials

Peak areas of each volatile compound recovered from different substrates and water were compared to peak areas of reference compounds directly subjected to thermal desorption and GC/MS analysis (i.e., in Table 1, peak area of 100 ng directly thermal-desorbed and analyzed compound was defined as 100 %). Similarly, in experiments using higher concentrations of volatiles, peak areas of compounds recovered from soil were compared to peak areas of reference compounds directly subjected to GC/MS (Table 3). In recovery experiments with sugars, samples were extracted, lyophilized, derivatized, and analyzed in an analogous manner to the samples of sugars obtained from substrates. A final volume of 1 ml was obtained after derivatization, of which 1 μl was subjected to GC/MS.

### Statistics

We used the statistics software ‘R’ (version 2.15.1, R Development Core Team 2012). Peak areas detected on contact and protected PDMS within one compound and substrate category (Table 1) were compared by Wilcoxon signed-rank tests (*P* ≤ 0.05). Substrate-specific recovery rates (Tables 1 and 2) were determined by Kruskal-Wallis *H-*test and *post-hoc* testing with Mann–Whitney tests including Bonferroni correction (*P* ≤ 0.05).

## Results

We developed a new tripartite mesocosm for sampling volatiles on PDMS tubes combined with aqueous extraction of sugars. The upper mesocosm compartment contained substrate and the plant rhizosphere, the central compartment was a perforated PTFE disc that contained moist air, and the lower drainage compartment contained air and leached aqueous nutrient solution. Sand was used as a substrate in mesocosms, due to its inertness and low specific surface area (SSA). For several reasons, dandelion (*Taraxacum* sect. *ruderalia*) was chosen as a model plant to test the function of the mesocosm: It is a globally distributed weed growing in soils of different properties (Stewart-Wade et al. 2002). The root chemistry is multi-faceted; total extracts of roots are rich in secondary compounds of pharmacological interest (Williams et al. 1996).

### Compounds from Dandelion Mescocosm Rhizospheres

When sampling from the newly-developed rhizosphere mesocosms with undamaged *T.* sect*. ruderalia* plants, we detected 15 volatiles and 3 sugars in total (Table 4).

**Fig. 3:**
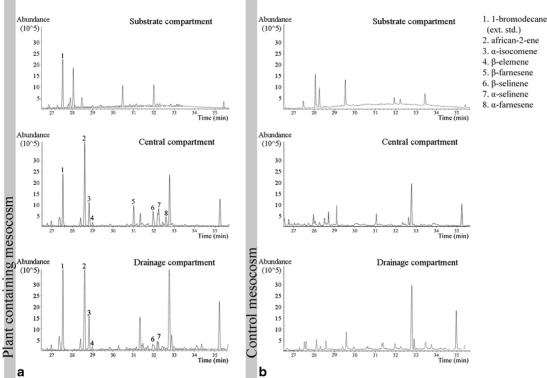
Detection of volatile rhizosphere chemicals collected in different compartments of the novel rhizosphere mesocosm (**a**) with substrate and *Taraxacum* sect. *ruderalia* plants and (**b**) without plants, but with substrate (= control); example TIC (total ion chromatograms) of volatiles. The percentage of dandelion compound peak areas of all detected compounds (unlabeled peaks were also found in plant-free controls) was highest in the central compartment (Mean ± SD: 23.5 ± 8.7 %) compared to substrate and drainage compartments (Mean ± SD: 5.1 ± 10.4 % and 19.6 ± 8.9 %)

Ten volatiles were identified as sesquiterpenes, and the sugars were identified as fructose, glucose, and sucrose. In the central compartment of the rhizosphere mesocosms, the highest number of volatiles was detected (overall 15 as compared to 12 and 8 in the substrate and drainage compartment, respectively). All volatiles present in the substrate and the drainage compartments were detected in the central compartment as well. The comparison of volatiles detected in the rhizosphere mesocosms to those sampled from plant-free control mesocosms allowed determining of “background” chemicals. In plant-containing mesocosms, the lowest level of substrate- or nutrient-solution derived compounds, and at the same time highest number and amount of plant-derived compounds, was found in the central compartment: 76.5 ± 8.7 % (mean ± SD) of the total peak area was annotated to substrate- or nutrient-solution background compounds as compared to 94.9 ± 10.4 % and 80.4 ± 8.9 % in the substrate and drainage compartment, respectively (example in Fig. 3).

Sugars were found exclusively in the substrate compartment but were not present in the drainage compartment. Quantities of sugars varied in average by less than 20 % between the two technical replicates (i.e., substrate samples of one mesocosm) and quantities of volatiles varied by less than 10 % between the three technical replicates (i.e., pieces of PDMS tubing in one mesocosm compartment). The variance among biological replicates was, however, relatively high (Table 4).

### Recovery Rates of Compounds Dependent upon Substrate Types in Vials

We determined recovery rates of commercially available volatiles from PDMS and sugars by aqueous extraction from plant-free substrates with different sorptive capacity (Tables 1 and 2), and from natural, untreated soil (Table 3). We used PDMS tubes that were left unprotected and PDMS tubes that were protected by a mesh, and thus, had no direct contact with the substrate. In general, adsorption capacities of substrates and recovery rates of volatiles did not follow a uniform trend.

When using unprotected PDMS tubes with direct contact to the substrate, the recovery of the volatiles methyl salicylate and 1-hexanol was better from sand compared to other substrates or even water (0.7 % of methyl salicylate and 0.4 % of 1-hexanol were recovered from sand, as compared to 0.1 % or 0 % from water, respectively). Other volatiles, such as α- and β-farnesene, α-pinene and butyl acetate were recovered best from the clay mineral vermiculite (36, 15, 15.2, and 2.8 %, respectively). Except for β-farnesene, recovery rates of these compounds were higher from vermiculite than from water. None of the compounds was extracted best from the processed (sieved and heated) fraction of field soil (Table 1) or from untreated soil (Table 3); however, extractability from the processed field soil fraction and sand was similar for six of the 12 tested volatiles on PDMS with contact to substrate. While 1-hexanol was neither recovered from the processed nor the untreated field soil when 100 ng were applied (Tables 1 and 3), the recovery of methyl salicylate was similar from the processed and untreated field soils (0.2 % for both, respectively). The recovered amounts of 1-hexanol and of the esters butyl acetate and hexyl acetate from field soil (Table 3) were marginal, even when high amounts were applied (1.1, 0, and 0 of the 5 μg applied were recovered, respectively). For some of the volatiles, the recovery rates from the processed fraction of field soil were much lower than from sand and vermiculite, i.e., for α- and β-farnesene, α-pinene, hexyl acetate, and 1-hexanol (0.9, 0.7, 7.2, 0.3, and 0 %, respectively, Table 1). The highest recoveries from natural, unprocessed field soil treated with 5 μg per compound were achieved for the ester methyl salicylate (44 ng); the aldehydes benzaldehyde and cinnamal (7.6 and 12.1 ng, respectively), and the monoterpenes α-pinene and linalool (61.2 and 5.5 ng, respectively) (Table 3).

When protecting the PDMS from contact to the substrate by a mesh cover, recovery rates were significantly improved for 9, 3, 7, and 2 out of the 12 volatiles applied to water, sand, vermiculite, and the processed soil fraction, respectively. For instance, the recovery rates of 1-hexanol and methyl salicylate were significantly improved for water, sand and vermiculite, and slightly improved for the processed soil fraction (Table 1). Protecting the PDMS tubes improved the recovery of α-pinene and butyl acetate from vermiculite (32.9 and 5.4 % as compared to 15.2 and 2.8 %, respectively) and α- and β-farnesene from the field soil fraction (from 0.9 and 0.7 to 2.1 % in both cases). However, the recovery of several compounds remained the same with and without the mesh cover, i.e., for α- and β- farnesene in vermiculite or all sesquiterpenoids in water. Some of the substances were even recovered at a reduced rate from protected compared to PDMS tubes with substrate contact (e.g., recovery of benzaldehyde and linalool from the processed soil fraction decreased from 5.1 and 6.9 % to 2.6 and 3.3 %, respectively).

**Fig. 4:**
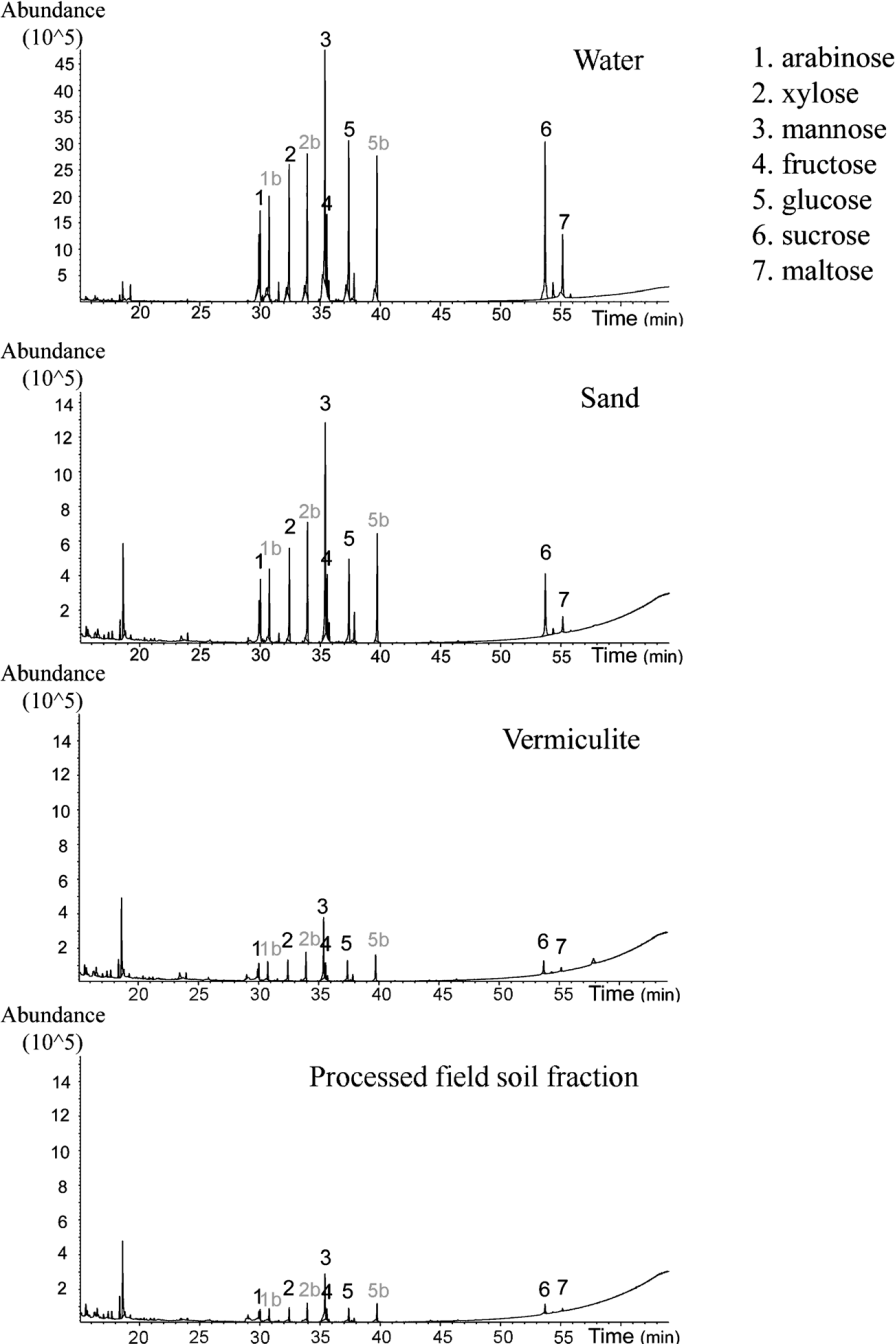
Determination of recovery rates of standard sugars applied to different substrates in a glass vial (10 μg of each sugar per vial). Example (total ion chromatograms) TIC of recovered compounds from sand, vermiculite, and a processed (sieved and heated) fraction of field soil compared to a standard mix in water (*N* = 4). Trimethylsilyl derivatives of arabinose, xylose and glucose were each represented by two peaks, a phenomenon that is commonly observed in gas chromatograms of silylated monosaccharides (Medeiros and Simoneit 2007)

In contrast to the complex pattern of volatile recovery rates in the substrates tested, the recovery rates of water-extracted sugars followed a clear uniform trend: best recovery was in all cases achieved from water, followed by sand; lowest recovery rates were obtained from vermiculite and the sieved and heated soil fraction (Table 2). Total ion current chromatograms of derivatized sugars extracted from different substrates are shown in Fig. 4.

## Discussion

This study demonstrates a convenient and non-destructive sampling technique for root-exuded metabolites. The combination of three rhizosphere compartments separated by permeable but inert material allows simultaneous determination of root-derived hydrophobic volatiles as well as non-volatile, water-soluble compounds, such as sugars. The recovery experiments indicate that the sampling method also is suitable for monoterpenes, ketones, and (aromatic) aldehydes. Hence, the set-up allows characterizing complex rhizosphere chemical profiles by use of silicone (PDMS) tubes for sampling of rhizochemicals. The robustness of the results (≤10 % variance among technical replicates within one sampling unit) matched the one reported by Kallenbach et al. 2014 (10–25 %) who sampled aboveground plant volatiles by using PDMS tubes. Volatile sampling from roots was most efficient when PDMS tubes were placed in the central mesocosm compartment in immediate vicinity of the rhizosphere, but without direct substrate-contact. Air- or water flows were not required, thus avoiding flooding, drying of the root zone, or root damage.

Plant roots were not obviously damaged, however, the degree of disturbance due to their removal from mesocosms has not directly been tested. To avoid removal of plants, it may be appealing to combine the application of PDMS with the use of a so-called rhizobox, as presented by Wenzel et al. (2001). Within the rhizobox, membrane systems separate growing roots and root hairs from surrounding substrates. Unfortunately, the establishment of substrate-specific rhizosphere bacterial communities (Marschner et al. 2004) and substrate-specific exudation of soluble and volatile compounds from roots (Rovira 1969) are suppressed in such rhizobox systems. To identify plant root-derived compounds that are potential belowground infochemicals, the here-presented experimental design offers the advantage that the plant roots remain in, or in close contact to the substrate. It, therefore, represents a step forward in approaching natural conditions when analyzing rhizosphere infochemicals.

Although ‘background’ components could not be classified due to their presence in plant-free but substrate and nutrient containing mesocosms, it cannot be excluded that ‘target’ components, listed in Table 4, are not exclusively plant-derived but also microbe-derived. If studies aim to unravel directly plant-derived compounds rather than microbially produced ones, the experiments could be conducted under sterile conditions thereby avoiding microbial degradation of compounds. For instance, application of Ag^+^ containing agents during collection of root exudates avoids microbial degradation after sampling (e.g., Gransee and Wittenmayer 2000).

Interestingly, we found that undamaged dandelion plants emit a unique volatile blend, even without herbivore damage (Table 4). Many of the detected sesquiterpenoids are rare in nature. In addition to volatiles, two monosaccharides (fructose, glucose) and one disaccharide (sucrose) were detected in dandelion rhizosphere samples. Their detection in rhizospheres under non-sterile conditions indicates their availability to soil biota including herbivorous insects, but the amounts were low, i.e., in the nanogram range. However, it has to be considered that only a small fraction of the total substrate volume (15 out of 1200 ml) per plant mesocosm was sampled for sugar analysis. Insects may detect extremely small amounts of volatile infochemicals in the nanogram range and below (e.g., review by Leitch et al. 2013 and references therein). Still, strong adsorption of root-released compounds to the substrate matrix (see below) may hinder diffusion and the establishment of gradients along which soil living animals may orient over a distance, or the attractiveness of some compounds may be masked by others (Reinecke et al. 2008). Thus, it remains to be determined whether the detected amounts of volatiles and sugars are sufficient to guide orientation of soil insects.

The mesocosm setup with plants grown in (non-sterilized) vermiculite also allowed detecting sugars in dandelion rhizospheres. The release of sugars from roots has so far been shown mainly for plants grown in sterile hydroponics (reviewed by Badri and Vivanco 2009; Dakora and Phillips 2002; Dennis et al. 2010). Sugars often account for the largest proportion of root-emitted compounds (Azaizeh et al. 1995) but are rapidly degraded with a half-life of about 0.5 to 2 h in non-sterile rhizospheres (Jones and Darrah 1996; Ryan et al. 2001) and have correspondingly barely been considered as potential infochemicals. Nevertheless, dandelion-feeding insect larvae have been shown to follow artificial gradients of sucrose in an arena assay (Eilers 2012). The release of sugars into the rhizosphere is a passive process, following a concentration gradient from plant tissue to soil solution (Jones and Darrah 1993). Rapid microbial degradation might counteract the establishment of a sugar gradient in natural soils. However, continuous release of sugars from roots might be used for close-range orientation of edaphon species to roots.

The recovery of various volatiles and sugars, some of which were present also in dandelion mesocosm rhizospheres (Table 4), was tested in water and various substrates applied to glass vials. The potential of the substrate matrix to clog the PDMS surface during volatile sampling also was assessed. Compounds belonging to the same chemical classes were surprisingly recovered to different extents from the same substrate, particularly when placing PDMS directly into the substrate (Table 1). Clear patterns of relationships between recovery rates, chemical structures, and substrate type became evident only for sugars, but not for volatiles. As a general trend, high volatility and low water-solubility (i.e., α-pinene, β-elemene, α- and β- farnesene) resulted in higher recovery from all substrates compared to compounds with high water-solubility, i.e., high polarity (e.g., butyl acetate and 1-hexanol). Recovery rates of volatiles that were applied to processed (sieved, heated) soil (Table 1) and to untreated field soil (Table 3) were low when compared to other substrates, and the amount of compounds recovered was close to the detection limit.

The low recovery of compounds may be due to several factors. First, the low amounts of PDMS material and short sampling duration of only 1 h in these recovery analyses might have caused the low recovery rates. The use of a higher amount of PDMS material (here: tube length of 3 mm), or extended sampling duration (here: 1 h, but 120 h in mesocosm experiments) may increase the recovery of sampled volatiles. Second, fast biological degradation of the chemicals may contribute to low recovery rates. The comparison of recovery rates from processed to untreated natural soil (see above) indicates that bacterial degradation could indeed be a major factor determining the availability of volatiles to both chemical analysis and other soil biota. Third, clogging of the PDMS surface by substrate particles may reduce recovery rates of rhizochemicals.

In the recovery experiments, PDMS tubes were either placed directly into the substrate, or substrate contact was avoided by protective gauze. For most compounds and substrates, recovery rates tended to be similar or higher when PDMS was protected by gauze. Similarly, the results from experiments in mesocosms indicate that some air space around the PDMS tubes may be required for the sorption of root volatiles in sufficient quantities for analysis, as best results were obtained when placing the PDMS tubes close to, but separated from the substrate, in the central compartment. The used mesh width of 100 μm in the recovery experiments may still allow smaller clay particles to interact or cover the PDMS surface, or clog the mesh itself. Hence, the gauze properties (material, mesh size, open area) further could be optimized and adapted to specific substrates.

Untreated natural soil differed in two aspects from other substrates. It contained organic matter, which was largely removed by sieving from the processed soil samples and had not been heated, i.e., microorganisms were present. As stated, bacterial degradation may be one reason for close to zero recovery from this substrate. On the other hand, numerous publications substantiate that the organic soil fraction adsorbs organic compounds of environmental concern such as herbicides and volatile hydrocarbons (e.g. Chiou et al. 1979; Shea 1989; Balseiro-Romero and Monterroso 2013). Extremely low recovery rates from untreated soil in experiments are in agreement with these reports and point at soil organic matter as an important sink for root-derived volatiles. If indeed adsorption to soil organic matter retains root-derived volatiles, this suggests another hypothesis. Living organic structures such as mycorrhizal networks act as transport routes for allelochemicals (Barto et al. 2011). Dead organic matter could in contrast act as a “storage facility” establishing lasting infochemical gradients that might inform foraging soil-inhabiting herbivores via taste or close range olfaction on plant roots in their vicinity.

In addition to an optimization and adaptation of the gauze properties and size of holes in the PTFE discs with regard to the used substrate, other studies investigating smaller or larger plants could adapt the mesocosm to the plant size. Furthermore, organisms interacting with the roots (e.g., rhizobacteria, mycorrhizal fungi, nematodes) could be added to the mesocosm to address questions on organism-dependent rhizosphere chemicals. Water soluble components other than sugars (i.e., amino acids, organic acids, flavonoids, glucosinolates) may be less prone to microbial degradation than sugars, and future studies need to unravel whether they occur not only in the substrate compartment, but also (primarily or in similar proportions) in the drainage compartment of a mesocosm. In addition to solvent extraction as performed here, non-volatile compounds may be purified and concentrated by ion-exchange chromatography (e.g., using XAD resin: Tang and Young 1982). We further expect that a simultaneous investigation of both volatile and water-soluble components also is feasible when plant rhizospheres are grown in sterile hydroponics; in this case, extraction and analysis protocols similar to those established here could be applied. Hence, the presented microcosm provides a tool that is easily adjustable to various experimental requirements and, thus, might be useful to address a wide range of future questions on plant rhizosphere chemistry.
